# Adherence to physical activity recommendations and barriers to physical activity participation among adults with type 1 diabetes

**DOI:** 10.1007/s11845-021-02741-w

**Published:** 2021-08-24

**Authors:** Mary Finn, Mark Sherlock, Sinead Feehan, Emer M. Guinan, Kevin B. Moore

**Affiliations:** 1 Department of Endocrinology, Tallaght Hospital, Dublin, Ireland; 2 Department of Nutrition & Dietetics, Tallaght Hospital, Dublin, Ireland; 3 School of Medicine, Trinity College Dublin, Dublin, Ireland; 4 Department of Endocrinology, Beaumont Hospital and Royal College of Surgeons in Ireland, Dublin, Ireland

**Keywords:** Accelerometry, Barriers, Cardiovascular disease risk, Physical activity, Self-reported questionnaires, Type 1 diabetes

## Abstract

**Background:**

Physical activity (PA) is important for those with type 1 diabetes (T1DM); however, accurate information on PA in people with T1DM is limited.

**Aims:**

This study assessed adherence to PA guidelines using both objective and subjective PA measures and evaluated the relationship between accelerometer-measured PA and cardiovascular disease (CVD) risk factors. Barriers to PA were also assessed.

**Methods:**

Using an observational cross-sectional design, PA was measured objectively over 7 days in 72 participants (34 males) using an accelerometer (ActiGraph) and subjectively using the International Physical Activity Questionnaire (IPAQ). Perceived barriers to PA were assessed using the Barriers to Physical Activity in Diabetes (type 1) scale. Multiple linear regression models assessed the influence of PA on HbA1c and CVD risk factors.

**Results:**

Mean age ± SD was 40.9 ± 12.9 years, diabetes duration was 18 ± 11.6 years, and HbA1c was 65 ± 14 mmol/mol /8.0 ± 1.3%. Twenty-three (32%) participants exercised according to PA recommendations as measured by an accelerometer. Sixty-nine (97%) participants reported meeting the recommendations as per the IPAQ. Those meeting recommendations (accelerometry) had a lower HbA1c (*p* = 0.001), BMI (*p* = 0.032), waist circumference (*p* = 0.006), and fat mass (*p* = 0.032) and a greater number of hypoglycaemic events (*p* = 0.004). Fear of hypoglycaemia was the strongest barrier to PA (mean 3.4 ± 2.0).

**Conclusion:**

The majority of participants failed to meet PA recommendations. Meeting the recommendations was associated with healthier CVD risk factor profiles. Individuals with T1DM possibly overestimate their PA using self-reported measures and require support and education to safely improve activity levels.

## Introduction

Among individuals with type 1 diabetes, cardiovascular disease (CVD) events occur at a younger age when compared to the general population [1]. Observational evidence, although limited, has shown that physical activity (PA) is associated with improved cardiovascular risk factors, including weight, blood pressure, lipid profile, and glycaemic control [2–5]. All of these studies have used subjective PA data collection measures (self-reported questionnaires), which are limited by certain biases, such as inaccurate memory and social desirability [6]. Only one available study has used an objective measure of PA to assess CVD risk benefits, and this study found that among adults with type 1 diabetes, 43% of women and 55% of men met the recommended PA level, which was associated with a significantly lower BMI, waist circumference, and percentage fat mass [7]. However, they found no benefits in terms of diabetes control. More studies using accelerometer-measured PA data are needed to thoroughly examine the influence of PA on glycaemic control and CVD risk factors. Accelerometers provide a more accurate estimate of PA and are both clinically feasible and cost-effective [6, 8].

Guidelines recommend that adults with type 1 diabetes should engage in 150 min or more of moderate-to-vigorous-intensity physical activity (MVPA), spread over at least 3 days/week, with no more than 2 consecutive days without activity [9]. Despite this recommendation, it would appear that the majority of adults with type 1 diabetes do not meet this level of PA [2, 5, 7, 10–13]. Several barriers to exercise in type 1 diabetes have been identified [14]. One of the main barriers to PA in children, adolescents [15], and adults [12, 14, 16] with type 1 diabetes is fear of hypoglycaemia.

Given the lack of available accelerometer data among this population, the primary objective of this study was to explore adherence to PA recommendations among adults with type 1 diabetes using an accelerometer and to evaluate the relationship between accelerometer-measured PA, glycated haemoglobin (HbA1c), and CVD risk factors. In addition, the barriers to PA among this population were explored.

## Participants and methods

### Study design and subjects

This study was an observational cross-sectional study. Inclusion criteria consisted of having a diagnosis of type 1 diabetes for greater than 1 year; on multiple daily injections of rapid and long-acting insulin or on insulin pump therapy; and aged ≥ 18 years. Participants were excluded if they were pregnant or if they had major diabetes complications or other chronic conditions meaning they were unable to ambulate without any limitations (e.g., severe peripheral or autonomic neuropathy). The Tallaght University Hospital/St. James’s Hospital Joint Research Ethics Committee approved the study protocol.

Recruitment took place at Tallaght University Hospital, Dublin, in two ways: Firstly, all previous participants (*n* = 150) who undertook our structured education programme for type 1 diabetes were invited to participate. Details of the study were posted to participants, and those interested in taking part, who met the inclusion criteria, were recruited (*n* = 52). Secondly, participants were randomly recruited (using an Excel computer random number generated list) at a weekly type 1 diabetes outpatient clinic over a 3-month period (*n* = 95), of whom 20 met the criteria and agreed to participate. In total, 72 people participated in the study.

Participants attended the diabetes centre at Tallaght Hospital and provided written informed consent prior to participation in the study. The following procedures were undertaken:

#### Accelerometry

Participants received the ActiGraph accelerometer (wGT3X-BT; ActiGraph LLC, Pensacola, FL, USA) to objectively measure PA. This is a small, lightweight accelerometer which has been extensively studied for its validity and reliability [17–20]. Participants were advised to maintain their normal PA level and to wear the accelerometer (over the right hip on an elasticated belt) for 7 days during waking hours. They were asked to remove it only when sleeping and bathing. All information was downloaded from the ActiGraph monitor to the ActiLife software. Data were recorded in 1-min epochs. Non-wear time was defined as a period of ≥ 90 min consecutive zeros with a spike tolerance of 2 min [21]. All participants had sufficient wear time and were included in the analysis (at least 4 days of at least 10 h per day).

To identify PA at the different intensities, count thresholds corresponding to the energy cost of the given intensity were applied to the data set [22]. Low-intensity PA was defined as between 100 and 2019 counts per minute (cpm); moderate intensity between 2020 and 5998 cpm; vigorous intensity ≥ 5998 cpm; and sedentary time < 100 cpm [22, 23].

Freedson Adult (1998) cut-points [24] were used to estimate the adherence to PA guidelines (defined as ≥ 150 min per week of MVPA in at least 10-min bouts). MVPA bouts of at least a 10-min duration (allows a drop time or non-compliant time of 2 min) were detected [24].

#### International physical activity questionnaire

Participants were asked to complete the International Physical Activity Questionnaire (IPAQ) following the week of PA monitoring. The self-reported PA questionnaire is the English language version of the long self-administered version of the IPAQ [25]. It captures PA performed in at least 10-min bouts over the previous 7 days, thereby capturing the same week as participants wore the accelerometer. It asks in detail about time spent walking and at moderate- and vigorous-intensity PA. Uniquely, the IPAQ also includes questions about time spent sitting as an indicator of sedentary behaviour. Adherence to PA recommendations was examined using the IPAQ categorical scoring, and those categorized as moderate or high activity were classified as meeting PA recommendations [26].

#### Barriers to PA

Participants completed the Barriers to Physical Activity in Diabetes (type 1) scale (BAPAD-1 scale), which was used to identify potential barriers to participating in PA [27]. Using this 11-item BAPAD-1 scale, participants rate barriers to PA on a scale of 1 to 7 (whether the item would keep them from practising regular PA over the next 6 months: 1, extremely unlikely, and 7, extremely likely).

#### Anthropometry and CVD risk factors

Participant’s height was measured using a stadiometer. Weight, BMI, and lean and fat mass were measured using the bio-electrical impedance analyzer (BIA) (Tanita body composition analyzer BC-420MA, Tanita Ltd, GB). Waist circumference was measured at the mid-point between the iliac crest and the lower rib margin, using a flexible, non-stretchable tape measure. Hip circumference was recorded at the widest part of the buttocks.

HbA1c, lipid, and renal profile were recorded from the institutional diabetes database. Blood samples were collected within 8 weeks of PA monitoring. Blood pressure readings were taken with the Dinamap PRO 300 blood pressure monitor. The presence of diabetic retinopathy was recorded.

During the week of PA monitoring, participants used the Freestyle Optium Neo (Abbott) to record all of their blood glucose (BG) readings. At the end of the week, the glucometers were downloaded and analyzed for average BG reading; BG standard deviation; number of BG readings; and number of hypoglycaemic events. Hypoglycaemia was defined as a BG of < 3.9 mmol/l [28].

Participants were also asked to keep a 7-day food diary (recorded all food, beverages, insulin boluses, and basal insulin throughout the day) and an exercise diary. Participants returned the accelerometer, IPAQ, and food and exercise diary by prepaid mail.

### Statistical analysis

Analyses were performed using IBM SPSS Statistics v 24. Study variable histograms were visually inspected for approximate normality prior to analysis. Descriptive analyses comprised number and percentages; mean and standard deviation; or median, 1st, and 3rd quartiles if substantial skew was observed. Descriptive statistics were stratified by whether the participants had undergone a structured education programme or not.

A range of variables were inspected for differences between those participants that met or did not meet recommended PA levels. These differences were also inspected in strata by gender, age, HbA1C above 8%, and structured education or not. No hypothesis tests were performed within strata due to small sample sizes and to avoid the effect of multiplicity on the type I error rate.

Multiple linear regression was performed to see if meeting recommendations for PA was associated with each of HbA1C (%), BMI, fat mass (kg), waist circumference (cm), and triglycerides (mmol/L) as dependent variables, controlling for gender, age, whether participants had undergone a structured education programme, and sedentary time (mean hours per day). Food diaries were analyzed using Nutritics (version 4.2).

## Results

Seventy-two adults (*n* = 34 males) with type 1 diabetes who fulfilled the inclusion criteria participated in the study. Baseline characteristics of the study population are shown in Table 1. Median age was 39 (IQR 30.5–47.7) years, diabetes duration was 18 ± 11.6 years, and HbA1c was 68.5 ± 12.6 mmol/mol (8.0 ± 1.28%). Median BMI was 26 (IQR = 23.2–30.2) kg/m^2^ with 57% of the participants being overweight or obese (28% obese). Analysis of food diaries revealed the percentage of energy from carbohydrate, protein, and fat was 43%, 19%, and 34%, respectively (Table 2). No correlations were found between nutrient intake and PA level or CVD risk. Those who had previously attended a type 1 structured education programme had a lower basal insulin (20 vs 29 units; *p* = 0.04) and took a greater number of boluses/day (4 vs 3; *p* = 0.01) than those who had not attended the programme. No differences were seen for CVD risk or PA levels.

**Table 1:** Characteristics of participants wearing the accelerometer: Total sample divided into those who received structured education and those who did not. Values expressed as mean ± SD, median (IQR), or number (*n*) (%). ^a^Mann-Whitney test; ^b^Student’s *t* test; ^c^Pearson’s chi-square test. Abbreviations: *HbA1c*, glycated haemoglobin; *LDL*, low-density lipoprotein; *HDL*, high-density lipoprotein. *Measures taken during the week of PA monitoring

Participants characteristics	No structured education (*n* = 20)	Structured education (*n* = 52)	Total sample (*n* = 72)	*p*-value
**Age (years)**	40 (IQR 24.7–58.3)	39 (IQR 31.7–45.7)	39 (IQR 30.5–47.7)	0.683^a^
**Diabetes duration (years)**	19.1 ± 12.31	17.5 ± 11.39	18 ± 11.58	0.606^b^
**Diabetes treatment**				
- MDI, *n* (%)	13 (65)	31 (60)	44 (61)	0.675^c^
- Insulin pump, *n* (%)	7 (35)	21 (40)	28 (39)	
**Smoking habit, *n* (%)**	5 (25)	10 (19)	15 (21)	0.589^c^
**Body mass index (BMI)**	26 (IQR 23.9–30.6)	26 (IQR 23.1–29.9)	26 (IQR 23.2–30.2)	0.920^a^
- Overweight, *n* (%) (BMI = 25.0–29.9 kg/m^2^)	5 (25)	16 (31)	21 (29)	0.689^c^
- Obese, *n* (%) (BMI > 30 kg/m^2^)	7 (35)	13 (25)	20 (28)	
**Waist circumference (cm)**	89 (IQR 81.2–105.0)	84 (IQR 77.4–95.2)	86 (IQR 78.3–95.6)	0.165^a^
**Hip circumference (cm)**	100 (IQR 95.0–112.5)	99 (IQR 96.1–106.9)	100 (IQR 95.7–108.4)	0.933^a^
**Body fat (%)**	26 (IQR 21.5–33.8)	29 (IQR 22.9–34.8)	28 (IQR 22.6–34.4)	0.342^a^
**Fat mass (kg)**	20 (IQR 16.4–24.6)	20 (IQR 17.2–27.6)	20 (IQR 16.7–27.3)	0.646^a^
**HbA1C (%)/(mmol/mol)**	8.4 ± 1.14/68.5 ± 12.62	7.9 ± 1.32/68.5 ± 12.62	8 ± 1.28/68.5 ± 12.62	0.132/0.133^b^
**Total cholesterol (mmol/l)**	4.6 ± 1.01	4.6 ± .68	4.6 ± .78	0.713^b^
**LDL (mmol/l)**	2.4 ± .94	2.4 ± .68	2.4 ± .75	0.944^b^
**HDL (mmol/l)**	1.6 ± .38	1.7 ± .46	1.7 ± .44	0.239^b^
**Triglycerides (mmol/l)**	1 (IQR .8–1.3)	1 (IQR .6–1.1)	1 (IQR .7–1.2)	0.149^a^
**Hypertension requiring treatment, *n* (%)**	6 (30)	11 (21)	17 (24)	0.429^c^
**Retinopathy, *n* (%)**	7 (35)	20 (38)	27 (38)	0.786^c^
**Total insulin (units)**	52 (IQR 40.8–59.9)	42 (IQR 32.1–59.4)	44 (IQR 32.7–59.4)	0.373^a^
- Basal	29 (IQR 22.0–35.0)	20 (IQR 16.0–28.0)	23 (IQR 16.7–30.1)	0.040^a^
- Bolus	22 (IQR 15.8–26.3)	22 (IQR 15.5–27.6)	22 (IQR 15.5–27.6)	0.875^a^
Number of boluses per day*	3 (IQR 2.9–3.4)	4 (IQR 3.0–4.3)	3 (IQR 3.0–4.1)	0.011^a^
Blood glucose variability (mmol/L)*	4.1 ± 1.17	3.9 ± 1.35	3.9 ± 1.30	0.548^b^
Average number of BG tests per day*	4 (IQR 3.6–6.0)	5 (IQR 4.0–7.6)	5 (IQR 3.7–6.9)	0.091^a^
Number of hypoglycaemic episodes*	3 (IQR 2.0–5.0)	3 (IQR 1.0–6.0)	3 (IQR 1.0–5.5)	0.720^a^

**Table 2:** Nutrient intake per day. Abbreviations: *%E*, percentage of energy

Nutrient	Mean	SD
Energy (kcal)	1778.0	432.9
Carbohydrate (g)	199.9	47.0
Carbohydrate (%E)	43.2	6.5
Protein (g)	84.6	26.6
Protein (%E)	19.2	4.7
Fat (g)	68.7	22.2
Fat (%E)	34.3	5.2
Alcohol (g)	9.5	14.3
Alcohol (%E)	3.4	4.9
Fibre (g)	18.2	4.9
Calcium (mg)	806.6	271.9
Iron (mg)	11.2	3.5
Vitamin D (µg)	2.5	1.5

### Adherence to PA recommendations

Twenty-three (32%) participants exercised according to PA recommendations as measured by an accelerometer (Table 3). Of the remaining 49 (68%) participants, the majority were well below the recommended level of PA, with 36 (50%) participants undertaking less than 60 min per week of MVPA in at least 10-min bouts.

**Table 3:** Differences between those adhering and not adhering to PA guidelines. Values are expressed as mean ± SD, median (IQR) or percentages. ^a^Mann-Whitney test; ^b^Student’s *t* test; ^c^Pearson’s chi-square test. Abbreviations: *MVPA*, moderate-to-vigorous-intensity physical activity; *HbA1c*, glycated haemoglobin; *BG*, blood glucose; *LDL*, low-density lipoprotein; *HDL*, high-density lipoprotein; *BMI*, body mass index

Variable	> 150 min (*n* = 23)	< 150 min (*n* = 49)	*p*-value
Total MVPA in 10-min bouts (min/day)	55.0 (IQR 41.3–68.3)	22.6 (IQR 11.5–30.7)	< 0.001^a^
Daily average of sedentary time (h/day)	8.6 ± 1.31	8.3 ± 1.65	0.328^b^
Male/female (%)	16.7 / 15.3	30.6 / 37.5	0.564^c^
Age (years)	32.8 (IQR 28.2–44.2)	40 (IQR 33.9–48.3)	0.111^a^
Diabetes duration (years)	14.9 ± 10.98	19.4 ± 11.70	0.132^b^
HbA1C (%)	7.3 ± 1.08	8.4 ± 1.24	0.001^b^
Total insulin (units)	39.7 (IQR 32.7–57.9)	47.1 (IQR 37.0–59.4)	0.230^a^
BG average (mmol/L)	8.1 ± 1.72	9.5 ± 2.12	0.007^b^
Number of hypoglycaemic episodes	5 (IQR 3.0–7.0)	2 (IQR 1.0–4.0)	0.004^a^
Total cholesterol (mmol/L)	4.5 ± 0.56	4.7 ± 0.86	0.424^b^
LDL cholesterol (mmol/L)	2.3 ± 0.58	2.5 ± 0.82	0.427^b^
HDL cholesterol (mmol/L)	1.8 ± 0.42	1.6 ± 0.44	0.180^b^
Triglycerides (mmol/L)	0.8 (IQR 0.7–0.9)	1.0 (IQR 0.7–1.3)	0.060^a^
Systolic blood pressure (mmHg)	136.5 ± 16.90	136.4 ± 13.51	0.968^b^
Diastolic blood pressure (mmHg)	75 (IQR 66.0–88.0)	75 (IQR 68.0–82.0)	0.858^a^
Weight (kg)	72.3 (IQR 65.1–79.1)	79.2 (IQR 67.9–96.1)	0.023^a^
BMI (kg/m^2^)	24.5 (IQR 22.5–26.3)	27 (IQR 23.4–31.4)	0.032^a^
Waist circumference (cm)	79.2 (IQR 76.0–87.1)	88.6 (IQR 80.4–101.0)	0.006^a^
Hip circumference (cm)	96.5 (IQR 93.3–100.9)	101.2 (IQR 96.7–112.3)	0.005^a^
% Body fat	26.8 (IQR 15.4–30.6)	28.6 (IQR 23.2–37.3)	0.122^a^
Fat mass (kg)	19.6 (IQR 11.2–21.5)	21.6 (IQR 17.2–31.3)	0.032^a^

More men than women met the PA guidelines (31% vs 15%). When stratified by age (18 to < 30, 30 to < 45, and 45 to 80 years), the amount of MVPA decreased with age (61 vs 51 vs 45 min/day) and the sedentary time increased with age (8.3 vs 8.6 vs 9.3 h/day). Participants spent the majority of their time sedentary (8.4 ± 1.6 h/day) or in light-intensity PA (4.4 ± 1.5 h/day), with a mean of 33.9 ± 22.2 min/day in MVPA (all minutes spent at MVPA). However, time spent in MVPA in at least 10-min bouts was only 60.5 (IQR = 16.5–168.3) min/week.

When we analyzed the self-reported data using the IPAQ, 69 (97%) of participants reported meeting the PA recommendations. Individuals reported less sedentary time (6.1 ± 2.4 vs 8.4 ± 1.6 h/day) and more moderate-intensity PA (187.0 ± 184.6 vs 31.9 ± 21.5 min/day) and vigorous-intensity PA (35.0 ± 64.2 vs 2.0 ± 3.9 min/day) than was evident from accelerometer data.

### CVD risk factors

Those meeting PA recommendations as measured by an accelerometer had a lower weight (72.3 kg vs 79.2 kg; *p* = 0.023), BMI (24.5 kg/m^2^ vs 27 kg/m^2^; *p* = 0.032), waist circumference (79.2 cm vs 88.6 cm; *p* = 0.006), hip circumference (96.5 cm vs 101.2 cm; *p* = 0.005), and fat mass (19.6 kg vs 21.6 kg; *p* = 0.032) than those who did not meet recommended targets (Table 3). HbA1c was significantly lower in those meeting PA guidelines (56 mmol/mol/7.3% vs 68 mmol/mol/8.4%; *p* = 0.001), as was glucometer BG average (8.1 mmol/l vs 9.5 mmol/l; *p* = 0.006) and individual BG standard deviation (3.5 mmol/l vs 4.1 mmol/l; *p* = 0.05). Finally, those meeting PA recommendations had a significantly greater number of hypoglycaemic events (5 vs 2; *p* = 0.004) during the week they wore the accelerometer.

Results of the multiple linear regression model are presented in Table 4. An inverse association was found between meeting PA recommendations and HbA1c (*R*^2^ = 14.5%; *P* = 0.001), BMI (*R*^2^ = 6.1%, *P* = 0.036), fat mass (*R*^2^ = 7.3%; *P* = 0.012), and waist circumference (*R*^2^ = 8.9%; *P* = 0.010).

**Table 4:** Associations of PA with CVD risk factors and glycaemic control. ^†^Adjusted for age, gender, participation in structured education, and sedentary activity. Independent variables: meeting exercise recommendations, ≥ 150-min moderate-to-vigorous physical activity per week; total time in 10-min bouts, total moderate-to-vigorous-intensity physical activity in 10-min bouts. Abbreviations: *HbA1c*, glycated haemoglobin; *BMI*, body mass index

Dependent variable	Independent variables					
	**Meeting exercise recommendations**			**Total time in 10-min bouts**		
	**Coefficient (95% CI)**	***P*-value**	**Partial *R***^**2**^	**Coefficient (95% CI)**	***p*-value**	**Partial *R***^**2**^
**HbA1C (%)**	− 1.08 (− 1.708, − 0.453)	0.001	14.5%	− 0.201 (− 0.346, − 0.056)	0.007	10.0%
**BMI (kg/m**^**2**^**)**	− 2.995 (− 5.788, − 0.201)	0.036	6.1%	− 0.628 (− 1.267, 0.01)	0.054	5.2%
**Fat mass (kg)**	− 7.748 (− 13.74, − 1.756)	0.012	7.3%	− 1.8 (− 3.16, − 0.441)	0.010	7.6%
**Waist circumference (cm)**	− 8.738 (− 15.333, − 2.143)	0.010	8.9%	− 1.92 (− 3.428, − 0.412)	0.013	8.3%
**Triglycerides (mmol/L)**	− 0.299 (− 0.604, 0.007)	0.055	5.6%	− 0.07 (− 0.137, − 0.003)	0.040	6.3%

### Barriers to PA

The mean BAPAD-1 total score was 2.4 ± 1.0 (range 1.0–5.4; rated on a scale of 1–7) (Table 5). Fear of hypoglycaemia was identified as the highest barrier score (3.4 ± 2.0). The other main barriers to PA included low fitness levels (2.9 ± 1.9), weather (2.8 ± 1.9), and loss of control over diabetes (2.7 ± 1.9). There was no association between BAPAD-1 total score and PA level measured by the accelerometer or diabetes control as measured by HbA1c. As percentage body fat and fat mass increased, there was a significant increase in BAPAD-1 total score (*p* = 0.004 and *p* = 0.026, respectively).

**Table 5:** Barriers to Physical Activity in Diabetes (BAPAD1) scale. Indicate the likelihood that each of these items below would keep you from practising regular PA during the next 6 months (1, extremely unlikely to 7, extremely likely)

Items	Mean score ± SD
1. The loss of control over your diabetes	2.7 ± 1.9
2. The risk of hypoglycaemia	3.4 ± 2.0
3. The fear of being tired	2.2 ± 1.5
4. The fear of hurting yourself	1.9 ± 1.5
5. The fear of suffering a heart attack	1.5 ± 1.1
6. A low fitness level	2.9 ± 1.9
7. The fact that you have diabetes	2.1 ± 1.6
8. The risk of hyperglycemia	2.1 ± 1.5
9. Your actual physical health status excluding your diabetes	2.5 ± 1.9
10. Weather conditions	2.8 ± 1.9
11. The location of a gym	2.2 ± 1.6
**Average score**	**2.4 ± 1.0 (range 1.0–5.4)**

## Discussion

Our study examined PA among a broad range of participants with type 1 diabetes and looked at the association between PA and CVD risk factors. This study has shown that only 32% of participants met the PA recommendations as measured by an accelerometer, and overall, there was a positive association between PA and HbA1c, and PA and body composition. The uniqueness of this study was the inclusion of an objective (accelerometer) measure of PA to examine the possible benefits on glycaemic control and CVD risk.

A number of other groups have measured PA using an accelerometer. A Canadian study using accelerometer data (SenseWear Pro 3 Armband) among 75 adults with type 1 diabetes found that 43% of women and 55% of men met the PA recommendations [7]. The higher levels of PA in this study could be due to the different accelerometers used and also the cut-points used to define physical inactivity. Two more recent studies have used the ActiGraph accelerometer to measure PA, as used in our study. A UK study showed that adults recently diagnosed with type 1 diabetes spent > 25% less time doing MVPA over the week when compared to healthy adults [11]. The other study from the USA found that adults with type 1 diabetes engaged in less MVPA than participants without diabetes and only undertook 37 min per week of MVPA in at least 10-min bouts [12]. This level was less than our finding of 60.5 min/week. To compare our findings to that of the general population that were living in a similar catchment area (Tallaght, Dublin 24) to our study participants, we looked at the 2014 HANA Survey [29]. This survey used self-reported PA questionnaires among 1082 individuals and found only 15.7% (*n* = 53/337) of respondents reported meeting the PA guidelines. This is far lower than our findings, which is interesting given that questionnaires can overestimate PA [30].

Our study is the first study to show an inverse association between glycaemic control (HbA1c) and accelerometer-measured PA data among adults with type 1 diabetes. This remained significant when controlled for several factors. This inverse association could have important clinical significance with those not meeting PA recommendations having a HbA1c well above clinical targets (8.4% vs 7.3%). In contrast to our findings, the only other available study that used accelerometer-measured PA data to assess CVD risk benefits did not see a difference in diabetes control with PA [7]. Compensation with diet and insulin adjustments in order to avoid exercise-induced hypoglycaemia could explain the absence of effect on glucose control [7]. However, consistent with their findings, we also detected an inverse association between PA and several CVD risk factors (weight, BMI, waist circumference, and fat mass).

It is possible that participants in our study underreported sedentary time and overreported moderate-intensity PA as 97% of our participants reported exercising to recommended levels as per the IPAQ. A previous study has shown that participants with diabetes may overestimate their PA levels [12]. Overestimation of self-reported PA may be caused by the desire to conform to social norm [6] and individuals misclassifying light-intensity PA as moderate-intensity PA [23].

Among our study participants, fear of hypoglycaemia was found to be the strongest barrier to PA. Other studies have also found hypoglycaemia as the most feared and frequently acute complication of PA [10, 12, 14–16]. Perceived risk of hypoglycaemia (measured by the BAPAD-1 score) was associated with spending less time in MVPA among individuals with diabetes [10, 12]. Our study found no significant correlations between the BAPAD-1 score and PA level as measured by an accelerometer; perhaps, a larger sample size could have detected associations.

Not only was fear of hypoglycaemia found to be the greatest barrier, but also incidence of hypoglycaemic events increased (*p* = 0.004) as MVPA increased. PA may increase the risk of hypoglycaemia due to an increase in insulin-stimulated glucose uptake into the muscles and an aerobic exercise-induced reduction of the blood glucose [31, 32]. From the literature, the majority of individuals with type 1 diabetes report a lack of practical advice for preventing exercise-induced hypoglycemia, with many feeling uneducated about required dose adjustments to insulin and carbohydrate around exercise [14, 16].

### Strengths and limitations

A major strength to this study is the use of an objective measure of PA which may be more reliable than self-reported PA measures. Also, our sample is representative of the general type 1 diabetes population (wide age and diabetes duration range and a mixture of participants on insulin pump therapy and multiple daily injections).

Our glucose data is from self-monitoring of blood glucose alone. The use of continuous glucose monitoring (CGM) in this study would have provided more comprehensive blood glucose data. Participants in this study volunteered to take part, which may have introduced bias by including those more engaged in habitual PA and omitting more sedentary individuals. Also, the authors cannot rule out the potential Hawthorne effect when using accelerometers as the study is reliant on participants engaging in a typical week of free-living PA. These analyses were cross-sectional in nature; thus, observed associations cannot prove a causal effect. A longitudinal study looking at the impact of PA on cardiovascular risk factors and outcomes is warranted.

In conclusion, the majority of participants with type 1 diabetes failed to meet PA recommendations. Exercise was positively correlated with weight, BMI, and HbA1c, highlighting the importance of PA among this group. Overestimation in PA is a serious concern, as it almost certainly results in individuals not getting sufficient exercise and therefore not realizing the associated improvements in CVD risk factors. Finally, diabetes-specific barriers do exist, specifically fear of hypoglycaemia, which result in poor uptake of exercise among this group. Our study results highlight practical gaps that need to be addressed through appropriate education on the impact of exercise on glucose variability and implementation of strategies to avoid exercise-induced hypoglycaemia. Development of educational tools (online resources/factsheets) based on the most recent guidelines on exercise management and type 1 diabetes [32] could be used to educate individuals, also directing individuals to the growing number of platforms where they can obtain information and advice (EXTOD; Runsweet; JDRF). Individuals should be encouraged to use wearable fitness tracking devices in order to validate their level of activity. Finally, support should be provided to those interested in using technologies such as CGM and closed-loop systems which have shown to contribute to increased time in range around exercise [33] and help prevent exercise-induced hypoglycaemia [34].

## Data Availability

All data is available on request.
